# Evaluation of antibacterial effects of different intracanal medicaments on *Enterococcus faecalis* in primary teeth: An in vitro study

**DOI:** 10.1002/cre2.718

**Published:** 2023-02-08

**Authors:** Bushra Munzer Shamma, Saleh Al Kurdi, Anas Rajab, Ettihad Abo Arrag

**Affiliations:** ^1^ Department of Pediatric Dentistry, Dental College Damascus University Damascus Syria; ^2^ Department of Organic Chemistry, Faculty of Pharmacy Syrian Private University Daraa Syria

**Keywords:** calcium hydroxide, chitosan, *Enterococcus faecalis*, propolis

## Abstract

**Objectives:**

Successful endodontic therapy is based on the reduction of infecting bacteria by cleaning, shaping, and disinfecting of the root canal system, thus the use of intracanal dressing is necessary for optimal success of root canal treatment. This study was designed to evaluate the effect of chitosan and propolis as intracanal medicaments against *Enterococcus faecalis* compared to calcium hydroxide in primary root canals.

**Material and Methods:**

Ninety‐six extracted primary second molars were collected. Teeth preparation was completed to size 30 K‐file. They were randomly divided into four groups; (A): chitosan, (B): propolis, (C): calcium hydroxide, and (D): control group (saline). The tooth specimens were inoculated with *E. faecalis*. Then, tested materials were applied for all groups in accordance to the groups each tooth belonged to. Following this, the bacterial colonies were counted after 24 h, 72 h, and 1 week of applying dressing materials and incubation. Finally, one–way analysis of variance and Fisher's least significant difference tests were used for statistical comparisons between the groups at a significance level of .05.

**Results:**

No statistically significant difference was found between groups A, B, and C for both 24 h and a week (*p* ≥ .05). Yet, a statistical difference between groups A, B, C, and D after 72 h and 1 week were seen (*p* ≤ .05).

**Conclusions:**

Chitosan and propolis medicaments were as effective as calcium hydroxide against *E. faecalis* in primary root canal treatment and might be considered as an alternative dressing material between treatment sessions.

## 1 INTRODUCTION

The long‐term success key of endodontic therapy is directly related to the reduction and elimination of the infecting bacteria inside root canal systems. (Attiguppe et al., 2017) This is achieved by cleaning and shaping root canals. (Öter et al., 2018) Therefore, multiple clinical methods of root canal disinfection and biofilm control during endodontic procedures were developed. However, the effectiveness of instrumentation on bacteria and their existence in dentinal tubules and lateral canal ramifications in primary teeth is limited (Attiguppe et al., 2017).

There are chances of pulp therapy failure in primary teeth even when chemo‐mechanical preparation is properly performed. (Navit et al., 2016) Therefore, interappointment intracanal medications were introduced. They help in infection control, preventing contamination between appointments, and case stabilization after the final obturation of the root canal system, leading to preferable treatment expectations (Mohammadi & Dummer, 2011).

*Enterococcus faecalis* are the most common bacterial species that have been detected in the failure of root canal treatment in both permanent and primary teeth. (Cogulu et al., 2007) *E. faecalis* are considered the most resistant strain in dental infection that must be removed in endodontic procedures (Attiguppe et al., 2017).

Calcium hydroxide has a wide range of antibacterial activity, but its effect is limited against some bacterial species, in particular *E. faecalis* and *Candida albicans* (Mustafa et al., 2012), that are resistant to the antibacterial action of calcium hydroxide (Haapasalo et al., 2000).

Chitosan is a natural biopolymer with a straight chain of cationic polysaccharide, which is derived from chitin—the main component of crustacean outer skeletons—by deacetylation (Tanikonda et al., 2014; Kmiec et al., 2017).

There are many amino groups linked to polysaccharide chain in chitosan that makes chemical reaction and the formation of salts with acids possible (Tanikonda et al., 2014).

High bioactivity is the main property of chitosan; that is why it makes it a promising new biomaterial in dentistry (Kmiec et al., 2017). In addition to that, it plays the role of wound dressing, hemostatic agent, drug delivery system, and antibacterial factor (Tanikonda et al., 2014; Husain et al., 2017).

Studies have shown the antibacterial activity of chitosan as a root canal irrigation; the effect of chitosan in reducing the bacterial population of *E. faecalis* when used to irrigate the root canals of permanent teeth was evaluated in studies (Jaiswal et al., 2017; Roshdy et al., 2019).

Propolis is a natural biocompatible resinous material, which is extracted from substances of plants collected by honey bees. Due to the presence of flavonoids in propolis, it has antimicrobial and anti‐inflammatory properties. Consequently, it has been used for oral and throat infection therapy, before being applied for dental caries management (Agrawal et al., 2017; Parolia et al., 2020).

Many studies have found that propolis is effective when used as an intracanal irrigant and a direct pulp capping agent (Jolly et al., 2013; Parolia et al., 2010). Furthermore, it was suggested as an intracanal medicament (Parolia et al., 2020).

This study was designed with the aim of evaluating the effect of both chitosan and propolis as an intracanal medicament against *E. faecalis* in primary root canal systems compared with standard medicament calcium hydroxide.

Null hypothesis (H_0_): There are no differences in antibacterial efficacy between study groups after (24 h, 72 h, and 7 days) of dressing application.

Alternative hypothesis (H_1_): There are differences in antibacterial efficacy between study groups after (24 h, 72 h, and 7 days) of dressing application.

## 2 MATERIALS AND METHODS

### 2.1 Study sample

The sample size was calculated according to a previous similar study (Attiguppe et al., 2017) using the G*Power software (v. 3.1) (Franz Faul, Universität Kiel, Germany). The significant level was set at 0.05, and the statistical power of the study was set at 95%. It was estimated that 20 teeth were required to demonstrate an effect size (0.72). Total sample size was raised by 20% (24 per group).

This is in vitro study consisting of 96 primary second molars extracted for orthodontic reasons. Inclusion criteria included the existence of two‐thirds of tooth root without any physiological or pathological resorption; moreover, they were not subjected to any treatment before extraction.

A radiograph of the extracted molars was performed, before and after opening the endodontic chamber.

Teeth were thoroughly washed with running water after extraction, cleaned with a gentle curettage to remove residual periodontal ligament, and scaled using U15 to remove any calculus. Then the teeth were soaked in Chloramine T 0.5% for a week with the purpose of disinfection (Rolland et al., 2007), then stored in sterile saline until they were used in this study. Samples were distributed randomly into four groups: chitosan, propolis, calcium hydroxide, and saline.

### 2.2 Teeth preparation

First of all, access openings were accomplished, and the working length was determined using a radiograph 1 mm shorter than the radiographic apex. Second, root canals were prepared by traditional technique up to the 30 K‐File (MANI, INC) size (10–5–20–25–30 sizes) (Attiguppe et al., 2017). During that, 1 ml of 2.5% sodium hypochlorite solution (Clorox® Performance) was used to irrigate the root canals. After completing the preparation, each root canal was irrigated with 5 ml of 2.5% sodium hypochlorite solution alternately with 5 ml of EDTA 17% (Produits Dentaires SA) and filled with 17% EDTA for 5 min to remove the smear layer (Zehnder, 2006). Third, apical foramen were sealed with flowable composite resin (TgDent), after which the teeth were placed in acrylic resin blocks to allow easier handling during the experiment. Then, teeth were sterilized with moist heat at 121°C for 30 min at a pressure of 15 pounds (lb; Öter et al., 2018).

### 2.3 Chitosan and propolis dressings preparation

Chitosan (≥75% deacetylated) was purchased from (Sigma‐Aldrich Chemicals), and propolis was purchased from (Sakka Amini).

For this purpose, 2 g of chitosan were dissolved in 100 ml of acetic acid (2%) (Sigma‐Aldrich Chemicals) with continuous stirring for 24 h till the formation of chitosan 2% (Ballal et al., 2010; Witedja et al., 2018).

Accordingly, 5 g of crude propolis were dissolved in ethyl alcohol (80%) in a ratio of (1:15), then the solution was evaporated under reduced pressure using a rotary evaporator (at 40–45°C). Afterward, the solution was filtered with (Whatman paper) (Healthcare GE) (Woo et al., 2015). Thereafter, approximately 1 g of sodium alginate 1% (Sigma‐Aldrich Chemicals) was added until the required thickness was reached.

### 2.4 Preparing bacterial suspension

*E. faecalis* was isolated clinically from the necrotic pulps of the patients treated in the department of pediatric dentistry, Damascus University, and the most resistant phenotype was determined using BD PHOENIX™ M100 (BD Company).

*E. faecalis* was cultured on Petri dishes with a nutrient medium (Mueller–Hinton *agar*) at 37°C for 24 h in a 5% CO_2_ incubator. Then, a bacterial suspension was formed and adjusted to (4.5 × 10^8^) colony‐forming units per mL (No. 1.5 McFarland Standard) using a BD PhoenixSpec™ nephelometer (BD Company). In the following stage, 50 μl of bacterial suspension (4.5 × 10^8^ [CFU/mL]) was applied into the prepared root canals using a sterile micropipette (Öter et al., 2018). Meanwhile, the suspension was activated in the canal using a sterile file size of 20 for the same period of 1 min for each sample. Later, all the samples were incubated at 37°C for 24 h under aerobic conditions (Öter et al., 2018).

After that, bacterial swabs were taken from five random samples and were cultured for confirmation that bacterial colonies were grown inside the root canal before applying the tested medicaments.

Then, samples were divided randomly into four groups to apply one of the tested materials. Randomization was performed using an online service (www.randomization.com).

Group (A) CH (*n* = 24): 2% chitosan was applied using a sterile syringe with disposable plastic needles to inject the dressing into the root canal, then lentulo spirals at low speed had been used.

Group (B) EEP (*n* = 24): 10% ethanol propolis extract dressing was applied using a sterile syringe with disposable plastic needles and lentulo spirals at low speed to inject the dressing into the canal.

Group (C) Ca (OH)_2_ (*n* = 24): calcium hydroxide (Produits Dentaires SA) dressing was applied after mixing with sterile saline solution in a volume ratio (1:1) using lentulo spirals at low speed.

Group (D) negative control group (*n* = 24): without applying any dressing in root canals.

Each canal was filled with its dressing before sealing the access cavity with glass ionomer cement (Fuji IX GP, GC Corporation). Finally, specimens were incubated at 37°C/CO_2_ 5% environment.

### 2.5 Evaluation of antibacterial effects

In this stage, bacterial colonies were counted after 24 h, 72 h, or 7 days of application of the dressing. Another factor to be considered is that the study groups were divided into three subgroups according to periods of intercanal medicament appliance:

1. 24 h (*N* = 32): (A1 = 8, B1 = 8, C1 = 8)
2. 72 h (*N* = 32): (A2 = 8, B2 = 8, C2 = 8)
3. 7 Days (*N* = 32): (A3 = 8, B3 = 8, C3 = 8)

**Figure 1:**
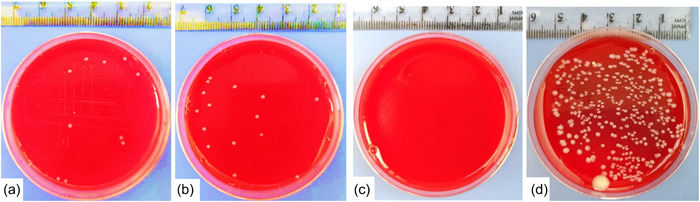
Growth of *Enterococcus faecalis* after 1 week of disinfection with respective test material (a: Chitosan, b: Propolis, c: Calcium hydroxide Ca(OH)_2_, d: Saline).

Root canals were rinsed with 1 ml of sterile saline, and the solution was left inside the canals for 1 min, with a peripheral filing using a sterile H File for 1 min also. Then, a paper point was inserted over the entire length of the canal for 60 s to absorb the contents of the canal. Next, the paper point was transferred to a sterile tube containing 2 ml of sterile saline; the paper point swab was repeated three times for each root canal to mimic its microbial state of it. Later, tubes with the paper points were shaken using a vortex device for 1 min to ensure the homogeneity of the solution, then 20 μl of the solution were taken by a sterile micropipette and inoculated on blood agar plates, before incubation with the same conditions as before for 24 h (Attiguppe et al., 2017; Camacho‐Alonso et al., 2017; Öter et al., 2018). In the end, bacterial colonies (colony forming units/ml) CFU/mL were calculated (Figure 1) and converted to logarithmic numbers to facilitate statistical analysis.

### 2.6 Statistical analysis

Data were coded and entered into the computer, statistical tests were conducted using SPSS software (17 version) (IBM). A *p*‐value < .05 was considered significant and had a confidence level of 95%. Data were summarized using mean with standard deviation for variables.

After using Kolmogorove–Smirnov test to determine if the data were molded by a normal distribution, the test showed that data after 24 h and 72 h are distributed normally. Therefore, to study the differences between experimental groups one way analysis of variance test was utilized at 0.05 significant level. While the data after 7 days are not distributed normally. Therefore, Kruskal–Wallis test was used to determine if there was a statistically significant difference between groups.

Subsequently, Fisher's least significant difference test was carried out to emphasize the differences between binary groups.

Wilcoxon test was carried out to study the significant bilateral differences in the mean bacterial colony units between the two stages before and after (24 h, 72 h, and 7 days) of applying the dressing.

## 3 RESULTS

**Figure 2:**
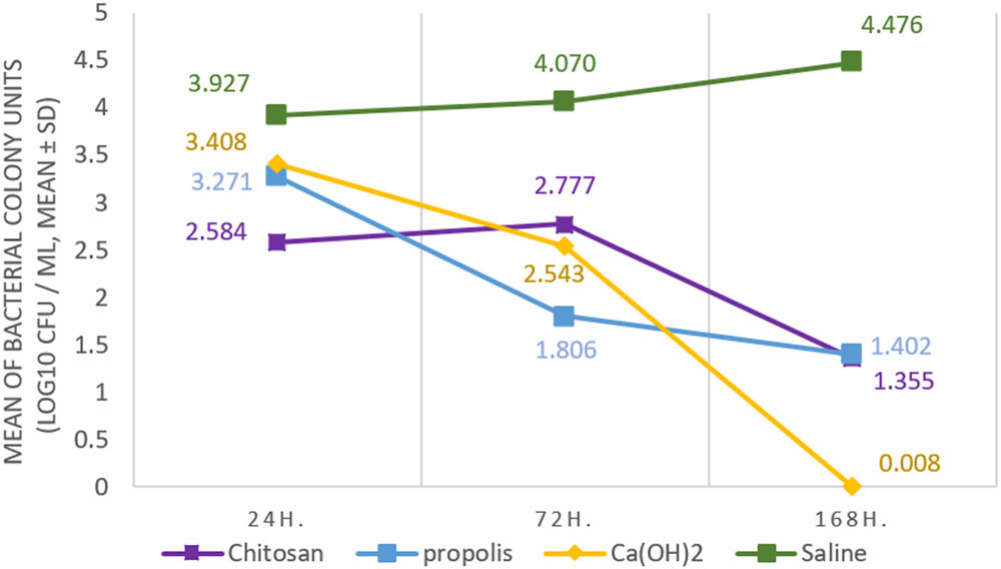
Mean of the bacterial colony units (log 10 CFU/mL, mean ± SD) in study groups during the studied time periods.

**Figure 3:**
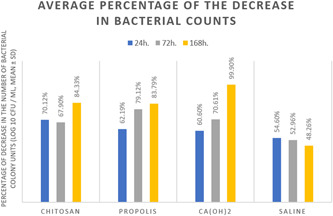
The average percentage of decrease in the number of bacterial colony units (log 10 CFU/mL, mean ± SD) for each studied dressing, where 1 log10 bacterial reduction equals 90%.

The descriptive statistics of the mean, standard deviation, and percentages of the decimal logarithm (log 10) of bacterial counts after disinfection procedures is shown in Figures 2 and 3.

The control group revealed the highest number of bacteria (mean 3.927 ± 0.936 log10 CFU/ml). Chitosan, propolis, and calcium hydroxide exhibited similar antibacterial effect after 24 h (2.584 ± 1.56, 3.271 ± 0.96, 3.408 ± 0.28 log10 CFU/ml) and 1 week (1.355 ± 1.25, 1.402 ± 0.99, 0.008 ± 0.01 log10 CFU/ml) respectively, without any statistically significant differences between them (*p* > .05) (Table 1).

**Table 1:** Shows results of the Fisher's LSD test to study the significance bilateral differences in the mean log 10 of bacterial colony units (log10 CFU/mL, mean ± SD) between the groups after (24 h, 72 h, and 7 days) of dressings applied.

	(I) Groups	(J) Groups	Mean difference (I–J)	Std. Error	Sig.
24 h	Chitosan	Propolis	−.686625	.479555	.161
		Calcium hydroxide	−.823875	.479555	.095
		Saline	−1.342750*	.479555	.008*
	Propolis	Calcium hydroxide	−.137250	.479555	.776
		Saline	−.656125	.479555	.180
	Calcium hydroxide	Saline	−.518875	.479555	.287
72 h	Chitosan	Propolis	.970375*	.462225	.043*
		Calcium hydroxide	.234125	.462225	.616
		Saline	−1.293125*	.462225	.008*
	Propolis	Calcium hydroxide	−.736250	.462225	.120
		Saline	−2.263500*	.462225	.000*
	Calcium hydroxide	Saline	−1.527250*	.462225	.002*
7 Days	Chitosan	Propolis	−.047250	.747940	.950
		Calcium hydroxide	1.347125	.747940	.080
		Saline	−3.121375*	.747940	.000*
	Propolis	Calcium hydroxide	1.394375	.747940	.071
		Saline	−3.074125*	.747940	.000*
	Calcium hydroxide	Saline	−4.468500*	.747940	.000*

Abbreviations: CFU, colony forming unit; LSD, least significant difference.

* The mean difference is significant at the .05 level.

After 72 h, propolis (1.806 ± 1.03 log10 CFU/ml) and calcium hydroxide (2.543 ± 0.31 log10 CFU/ml) demonstrated similar antibacterial effects with no statistically significant difference (*p* > .05). On the contrary, the antibacterial effect of propolis was higher than Chitosan (2.777 ± 0.86 log10 CFU/ml) with a statistically significant difference (*p* < .05). While, there is no statistically significant difference (*p* > .05) between the efficacy of chitosan, propolis, and calcium hydroxide after 1 week of application (Table 1).

Chitosan, propolis, and calcium hydroxide dressings administrated a significant decrease in the bacterial count, with a statistically significant difference (*p* < .05) in comparison to the bacterial counts before applying the dressing in all studied periods (Table 2).

**Table 2:** Shows results of the Wilcoxon test to study the significance bilateral differences in the mean log10 of bacterial colony units (log10 CFU/mL, mean ± SD) between the two stages before and after (24 h, 72 h, and 7 days) of applied the dressing in study groups, the mean difference is significant at *p* value < 0.05.

Bilateral comparisons of bacterial colony units between the two stages before and after		Chitosan	Propolis	Ca(OH)_2_	Saline
		*p*‐Value			
Before	24 h	.012*	.012*	.012*	.012*
	72 h	.012*	.012*	.012*	.012*
	1 week	.012*	.012*	.011*	.012*
After 24 h	72 h	.779	.012*	.012*	.575
	1 week	.161	.025*	.012*	.327
After 72 h	1 week	.012*	.575	.012*	.484

Abbreviations: CFU, colony forming unit; SD, standard deviation.

* The mean difference is statistically significant.

The average percentage of decrease in the number of bacterial colony units (log10 CFU/ml) for each studied dressing is shown in Figure 3, where 1 log10 bacterial reduction equals 90%.

## 4 DISCUSSION

Residual facultative anaerobic bacteria in the root canal system are the main cause of endodontic failure, and *E. faecalis* are the most prevalent bacteria (Cogulu et al., 2007). This bacterium is able to survive in dentinal tubes and even resist calcium hydroxide alkalinity (Stuart et al., 2006).

Although this study did not evaluate the effect of the studied intracanal medicaments on the biofilms of *E. faecalis*, it evaluated the effective duration of this intracanal medicament, as they were tested in three different time periods, to define the optimum effectiveness which might be considered beneficial in clinical practice of root canal disinfection (Neelakantan et al., 2007).

The results of this study showed reduction of bacterial count after applying the dressings compared to the bacterial count before with a statistically significant difference, in studied groups (Chitosan, EPE, Ca(OH)_2_) and during the three time periods studied.

The antibacterial ability of chitosan was higher than propolis and calcium hydroxide after 24 h of applying the dressing with no statistically significant difference; yet, with statistically significant difference compared to the negative control group.

The mechanism of antibacterial action of chitosan is attributed to the interaction between positively charged chitosan molecules and negatively charged bacteria cell membranes; in addition to that, leakage of intracellular components (proteins, nucleic acids, and glucose), which leads to bacterial growth inhibition (Goy et al., 2009). Another mechanism proposed is the permeation of chitosan into bacterial nuclei and binding with bacterial DNA that leads to inhibition of the mRNA and protein synthesis (Goy et al., 2009).

Jaiswal et al. (2017) mentioned that chitosan and propolis as irrigants had similar antibacterial effects against *E. faecalis*. (Jaiswal et al., 2017). Consequently, both chitosan and propolis are effective in bacterial elimination whether they were used as an irrigant material or as an intracanal medicament between endodontic treatment sessions.

After 72 h propolis shows the highest antibacterial activity followed by calcium hydroxide and chitosan.

Antibacterial activity of Ethanol Propolis Extract was conducted by flavonoids, aromatic components (acid kafeat), and phenol components. These active components of propolis will prevent cell division that will in turn inhibit the growth of bacteria (Lotfy, 2006; Netíková et al., 2013). In addition to that, the active ingredients damage the cell wall of bacteria and cytoplasmic membrane, inhibiting both bacterial protein synthesis and enzyme activity (Boyanova et al., 2006).

Ahangari et al. (2012) showed that the propolis extract 30% was similar to calcium hydroxide against *E. faecalis* strains after 72 h, 1 week, and 1 month of applying. These results are in accordance with those obtained in this study, although the concentration of propolis used by Ahangari et al. (2012) was 30%, which is higher than 10% concentration used in this study. In addition to that, the concentration of bacterial suspension (1.5 × 10^8^ CFU/ml) was lower than the one determined in this study.

In an in vitro study carried out by Sabarathinam and Muralidharan (2018) showed that chitosan has antibacterial activity less than calcium hydroxide against *E. faecalis* (chitosan has decreased 24% of the bacterial load while calcium hydroxide reduced 55% of the bacterial load after 72 h of intracanal medicament application. The previous study does not agree with the results of the present study that indicates the similar antibacterial activity of chitosan and calcium hydroxide after 72 h. Even though Sabarathinam and Muralidharan (2018) study was carried out on single‐root permanent teeth and the bacterial suspension used was (1.5 × 10^8^ CFU/mL) that are lower than that used in this study.

Antibacterial ability of calcium hydroxide was higher than chitosan and propolis after 1 week of applying the dressings with no statistically significant difference.

Long‐term application of calcium hydroxide has bactericidal properties, as it can lead to damaging bacterial cytoplasmic membranes that in turn lead to cell death (Mohammadi & Dummer, 2011; Siqueira & Lopes, 1999). Also, chitosan and EPE are bactericidal, (Goy et al., 2009; Djauharie & Kemala, 2017) which can explain the high effect of all three intracanal medicaments after an appropriate duration of application, up to a week of it.

These results are in accordance with Parolia et al. (2020) study, which found that chitosan was less effective than calcium hydroxide and propolis against *E. faecalis* after 1 week of dressing application. The concentration of chitosan in Parolias’ study was 0.2%, which was conducted on permanent teeth, *E. faecalis* were incubated for 21 days, and the bacterial suspension was used at (1.5 × 10^8^ CFUs/mL), yet the results of both studies were similar, which can lead to conclude that the effect of calcium hydroxide is higher in comparison to chitosan, even when the concentration of the last one was increased here (Abhishek Parolia et al., 2020).

When it comes to the study of Hussein et al. (2019), they indicated that chitosan and calcium hydroxide had a similar antibacterial effect after 7 days and 14 days of application as an intracanal medicament (Hussein et al., 2019). Likewise, Madhubala et al. (2011) exhibited that the effect of propolis against *E. faecalis* was 100% following 7 days of application. Both displaying similar results to the present study.

It was noted that there were statistically significant differences between the three time periods of application of calcium hydroxide dressings, as the greatest effectiveness was noted after 1 week of application. Although *E. faecalis* are resistant and able to survive up to a pH of 11.5, calcium hydroxide shows a pH ranging from 8.1 to 11.8 after 72 h of applying the dressing, which might be sufficient for bacterial elimination. It was seen that antibacterial activity of calcium hydroxide depends mainly on releasing hydroxyl ions and raising the pH locally (De Andrade de Andrade Ferreira et al., 2004). Likewise, the success of calcium hydroxide as an interappointment medicament against endodontic bacteria prompted its use as root canal filling material medicament prompted its use in sealer cement formulations for primary teeth (Vitapex) (Mohammadi & Dummer, 2011; Dean & Jeffrey, 2016).

Awawdeh et al., in 2009, indicated that the application of calcium hydroxide for less than a week does not reach the aimed disinfection of the root canal system, as long as the effectiveness of it against *E. faecalis* depends on the long‐term application of the dressing (Awawdeh et al., 2009). The same was seen in this study for calcium hydroxide and for the compared dressing materials.

On the other hand, a significant reduction in the number of colonies was found for the propolis extracts on Day 1, 3, and 7, whereas the effect of propolis was highest after 72 h of application but with no significant differences compared with the application for 1 week.

The total flavonoid content of ethanol propolis extract has a tendency to increase with ethanol concentration raised up to 70%. Also, it was observed that propolis output increased with temperature elevation (Woo et al., 2015). Here, propolis was extracted by an ethanol concentration of 80% at room temperature and demonstrated enough antibacterial properties.

In in vitro studies carried out by Saha et al. (2015), and Vasudeva et al. (2017), they manifested that propolis was more effective than calcium hydroxide against *E. faecalis*. Its antibacterial effect increased with a longer period of application reaching its peak on the fifth day (Saha et al., 2015; Vasudeva et al., 2017).

Meanwhile, chitosan showed similar antibacterial effects in the three study periods with no statistically significant differences. This can be reflected in clinical practice where the antibacterial effect is time‐dependent. However, further studies are necessary on the antibacterial behavior of chitosan in vivo conditions.

## 5 LIMITATION OF THE STUDY

Although this laboratory study is accurate, it is not completely similar to the clinical situation, and there are many factors that play a role in bacterial leakages, such as the marginal seal and the quality of various restorative materials, in addition to the level of oral care in the child and the child's immunity in general, as the effectiveness of the studied intra‐canal dressings clinically validated through randomized controlled clinical trials and long‐term follow‐up use of these dressings in endodontic treatments of primary teeth needs to be clinically proven, to confirm the antibacterial effect.

## 6 CONCLUSION

Complete eradication of bacteria in root canal system seems as impossible while decreasing of the bacteria to the maximum, might aid in the long‐term success of the endodontic treatment. Under the limitations of this in vitro study, chitosan, and propolis were as effective as calcium hydroxide against *E. faecalis* in the period of 1 week of dressings application.

## AUTHOR CONTRIBUTIONS

**Bushra Munzer Shamma**: Conception and design of study, intellectual content, methodology, acquisition of data, drafting, and writing the manuscript. **Saleh Al Kurdi**: Data curation, data analysis, and revising of the manuscript. **Anas Rajab**: Supervision, investigation, and revising the manuscript. **Ettihad Abo Arrag**: Supervision, investigation, and revising the manuscript.

## CONFLICTS OF INTEREST STATEMENT

The authors declare no conflicts of interest.

## ETHICAL STATEMENT

The study was in vitro conducted in the department of pediatric dentistry, faculty of dentistry, Damascus University and Al Mouwasat hospital, Damascus, Syria. Ethical clearance (ID: 102/M.S/2020) was obtained for conducting the study by the ethical review board of the faculty of dentistry and Damascus University, Syria.

## Data Availability

The data that support the findings of this study are available from the corresponding author upon reasonable request.
